# Temperature Compensation Method Based on Bilinear Interpolation for Downhole High-Temperature Pressure Sensors

**DOI:** 10.3390/s24165123

**Published:** 2024-08-07

**Authors:** Yizhan Shu, Chenquan Hua, Zerun Zhao, Pengcheng Wang, Haocheng Zhang, Wenxin Yu, Haobo Yu

**Affiliations:** College of Control Science and Engineering, China University of Petroleum (East China), Qingdao 266580, China

**Keywords:** high temperature, temperature compensation, piezoresistive pressure sensor, bilinear interpolation, least square

## Abstract

Due to their high accuracy, excellent stability, minor size, and low cost, silicon piezoresistive pressure sensors are used to monitor downhole pressure under high-temperature, high-pressure conditions. However, due to silicon’s temperature sensitivity, high and very varied downhole temperatures cause a significant bias in pressure measurement by the pressure sensor. The temperature coefficients differ from manufacturer to manufacturer and even vary from batch to batch within the same manufacturer. To ensure high accuracy and long-term stability for downhole pressure monitoring at high temperatures, this study proposes a temperature compensation method based on bilinear interpolation for piezoresistive pressure sensors under downhole high-temperature and high-pressure environments. A number of calibrations were performed with high-temperature co-calibration equipment to obtain the individual temperature characteristics of each sensor. Through the calibration, it was found that the output of the tested pressure measurement system is positively linear with pressure at the same temperatures and nearly negatively linear with temperature at the same pressures, which serves as the bias correction for the subsequent bilinear interpolation temperature compensation method. Based on this result, after least squares fitting and interpolating, a bilinear interpolation approach was introduced to compensate for temperature-induced pressure bias, which is easier to implement in a microcontroller (MCU). The test results show that the proposed method significantly improves the overall measurement accuracy of the tested sensor from 21.2% F.S. to 0.1% F.S. In addition, it reduces the MCU computational complexity of the compensation model, meeting the high accuracy demand for downhole pressure monitoring at high temperatures and pressures.

## 1. Introduction

Downhole pressure high-accuracy measurements at high temperatures and high pressure are critical for monitoring dynamic changes in pressure or pressure profiles during oil and gas exploration & production [1,2]. However, due to the harsh environment of high temperature, high pressure, strong corrosion, and minor space, it is difficult for common sensors to measure and monitor downhole pressure [3]. The resonant quartz pressure sensor is utilized for downhole pressure measurement due to its extremely high precision of 0.01% F.S., as well as its high level of stability and reliability in hard environmental conditions [4]. However, its high cost, extensive maintenance requirements, and inadequate reliability limit its use in downhole applications. The optical fiber sensor is the best contender for down-hole pressure monitoring due to its low cross-sensitivity, high resolution, and distributed measurement [5]. However, its high cost and low reliability prevent its widespread application in oil and gas wells.

Silicon piezoresistive pressure sensors are employed to monitor downhole pressure under high temperatures and high pressure due to their high accuracy, high level of stability, particularly minor size, and low cost [6]. Silicon-based piezoresistive sensors suffer from a sharp drop in measurement accuracy and reliability due to the change in resistivity of silicon material with temperature, and particularly high and highly variable downhole temperatures lead to a large bias in pressure measurement under downhole high temperature and high pressure. An on-chip integrable piezoresistive pressure sensor with 3-layer or 4-layer structures was proposed to enhance temperature robustness, and it was designed without any additional processes and therefore enables to compatible to the CMOS devices with low-cost and suitable for IoT (Internet of Things) applications. However, it only addresses the issue of high-temperature reliability, not the effect of temperature on measurement accuracy [7,8].

Therefore, in order to maintain high accuracy and long-term stability for downhole pressure monitoring in high temperatures, an appropriate temperature compensation is required [9,10]. Previous research on temperature compensation for silicon-based piezoresistive sensors can be loosely classified into two categories: hardware self-compensation and software compensation methods. Hardware self-compensation solutions often include adding passive components or extra circuits directly into the sensor package. These components are specifically intended to cancel out temperature-induced changes in sensor output. For example, resistive compensation employs extra piezoresistors with a Negative Temperature Coefficient of Resistivity (TCR) for temperature compensation [11,12], and the built-in compensation technique eliminates the need for an expensive and time-consuming calibration process required for each sensor inside a fabricated batch. By including passive resistors in the sensor circuit, the temperature properties of the resistors can be leveraged to mitigate the effects of sensor temperature drift [13]. A silicon on-insulation (SOI) material and a standard MEMS process are used in pressure-sensitive chip fabrication, and high-temperature electronic components are adopted in the temperature-compensation and signal-conditioning circuits. Although hardware self-compensation helps decrease temperature drift and non-linear errors, each sensor must have its own self-compensation circuits, increasing the complexity of circuit design [14].

Software temperature compensation approaches for piezoresistive pressure sensors use a correction model to mitigate the impacts of temperature variations and nonlinearity. Unlike hardware compensation methods that rely on built-in circuits, software algorithm compensation is accomplished using data processing techniques and a correction model, which is more flexible and adaptable. Polynomial fitting and lookup table-based compensation methods are the most basic temperature compensation algorithms [15], however they can only reach 1% F.S. measurement accuracy. In recent years, machine learning techniques such as deep neural networks (DNNs) and convolutional neural networks (CNNs) have begun to be used for temperature adjustment of piezoresistive pressure sensors [16,17,18,19]. Learning the complex sensor-environment interaction can considerably enhance compensation and accuracy. However, the complicated machine learning technique is difficult to execute with a high-temperature MCU and does not meet the real-time measurement requirements [20,21]. 

For downhole pressure monitoring in high temperatures, ambient temperature has a significant impact on the measurement accuracy of the silicon piezoresistive pressure sensor. These temperature coefficients differ from manufacturer to manufacturer and even vary from batch to batch within the same manufacturer. As a result, in order to provide high accuracy and long-term stability for downhole pressure monitoring in high temperatures, it is critical to perform high-temperature and high-temperature co-calibration on each sensor and suggest an appropriate temperature compensation method. A temperature compensation method based on bilinear interpolation for high-precision piezoresistive pressure sensors suitable for downhole high-temperature and high-pressure environments was proposed. After fitting by least squares and interpolating, the bilinear interpolation approach was presented to compensate for temperature-induced pressure bias, which is more easily implemented in MCU.

## 2. Temperature Effects on Accuracy of Piezoresistive Pressure Sensor

### 2.1. Piezoresistive Effect of Piezoresistive Pressure Sensor

**Figure 1:**
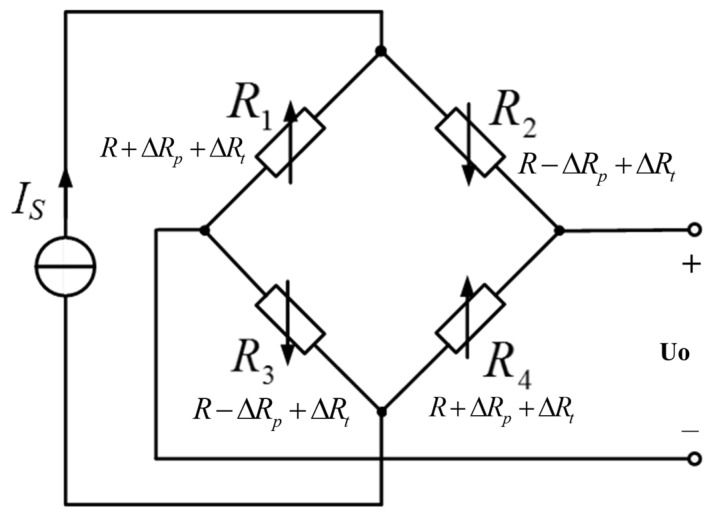
Schematic diagram of piezoresistive pressure sensor.

The silicon piezoresistive pressure sensor converts the stress applied to the silicon into a change in resistance due to the piezoresistive effect. It is often designed in a Wheatstone full-bridge configuration with four silicon piezoresistors, as shown in Figure 1. 

Ideally, when the pressure is zero and the temperature is the reference temperature *T_0_* at 25 °C, the four piezoresistors should have the same initial resistance *R*, as shown below.

(1) $$R_{1}=R_{2}=R_{3}=R_{4}=R$$

When a constant current source *Is* applied to the Wheatstone bridge, the sensor’s output voltage $U_{o}$ with loaded pressure *P* is as follows:

(2) $$\begin{array}{ll} U_{o} & =\frac{1}{2}I_{s}(R+\Delta R_{p}+\Delta R_{t})-\frac{1}{2}I_{s}(R-\Delta R_{p}+\Delta R_{t})\\ & =I_{s}\times \Delta R_{p}=I_{s}R\pi \sigma \end{array}$$

(3) $$R_{1}=R_{4}=R+\Delta R_{p}$$

(4) $$R_{2}=R_{3}=R-\Delta R_{p}$$

where, $\Delta R_{p}$ represents the change in resistance of the four piezoresistors when a pressure *P is* applied to the sensor, π is the piezoresistive coefficient of silicon, and σ is the stress applied to the silicon that corresponds to the pressure *P*.

Equation (2) indicates a linear relationship between $U_{o}$ and σ when both the current source Is and the piezoresistive coefficient π are constant. So the pressure P can be deduced by measuring $U_{o}$. However, the change in ambient temperature has a great influence on the four piezoresistors of the sensor and the following measurement circuits, which will affect the measurement accuracy of the pressure measuring system. For this reason, the effect of ambient temperature on the various components of this pressure measurement system is analyzed in detail below.

### 2.2. Analysis of Temperature Effects

Equation (2) reveals that in addition to the influence of pressure on the resistance of the Wheatstone bridge’s four piezoresistors, ambient temperature T has a considerable effect on resistance, silicon piezoresistive coefficient, stress, and current source [22].

(1) Temperature effects on resistance

The ambient temperature T has the same effect on the resistance of the four piezoresistors, as seen below:

(5) $$R^{\prime }=R+\Delta R_{t}=R(1+\alpha \Delta T)$$

The sensor’s output voltage $U_{o}$ can be represented as follows:

(6) $$U_{o}=\Delta R_{p}\times I_{s}=I_{s}R^{\prime }\pi \sigma =I_{s}R(1+\alpha \Delta T)\pi \sigma$$

where, α denotes the temperature coefficient of the pressure-sensitive resistor, ∆T is the change of ambient temperature, and T refers to the reference temperature T_0_ and reflects the change in resistance of the four piezoresistors when the ambient temperature changes by ∆T.

(2) Temperature effects on the piezoresistive coefficient

Furthermore, Equation (2) demonstrates that ambient temperature has a significant influence on silicon’s piezoresistive coefficient, as follows:

(7) $$\pi =\pi_{0}(1+\beta \Delta T)$$

where, π_0_ is the piezoresistive coefficient of silicon at the reference temperature *T*_0_ at 25 °C, and β denote the temperature coefficient of the silicon material.

(3) Temperature effects on the stress

Simultaneously, the change in ambient temperature will cause a residual stress between the support beam and substrate in addition to the applied pressure, as follows.

(8) $$\Delta \sigma =\frac{\left(\alpha_{s}-\alpha_{g}\right)E_{0}\Delta T}{1+\mu }$$

where, $\alpha_{s}$ and $\alpha_{g}$ represent the thermal expansion coefficients of silicon and PTFE glass respectively, μ denotes the Poisson ratio of the monocrystalline silicon, and $E_{0}$ is the temperature coefficient of silicon in Kelvin temperature.

By substituting Equations (7) and (8) in Equation (9):

(9) $$U_{o}=I_{s}R(1+\alpha \Delta T)\pi_{0}(1+\beta \Delta T)\left(\sigma +\frac{\left(\alpha_{s}-\alpha_{g}\right)E_{0}\Delta T}{1+\mu }\right)$$

Actually, due to the unavoidable manufacturing defects among the four piezoresistors, the additional offset component must be added to Equation (9), which is as follows:

(10) $$U_{o}=I_{s}R\pi_{0}(1+\alpha \Delta T)(1+\beta \Delta T)\left(P+\frac{\left(\alpha_{s}-\alpha_{g}\right)E_{0}\Delta T}{1+\mu }\right)+U_{o0}$$

Equation (2) shows that when the temperature is constant, the output $U_{o}$ is positively proportional to the pressure *P*. However, Equation (10) shows that changes in ambient temperature (∆T) also have a significant effect on this relationship by affecting the temperature coefficients, such as $\pi_{0}$, α, β, $E_{0}$, $\alpha_{s}$, $\alpha_{g}$, μ and $U_{o0}$, which will affect the measurement accuracy of the silicon piezoresistive pressure sensor. These temperature coefficients differ from manufacturer to manufacturer, from batch to batch from the same manufacturer, and even within the same batch from the same manufacturer. Because some production process parameters are confidential, these temperature coefficients in Equation (2) cannot be fully available. When the sensor is used for common temperature and low-accuracy applications, the temperature effects are typically minimal. However, in order to achieve high accuracy and long-term stability for downhole pressure monitoring in high temperatures, a number of calibrations must be performed on a high-temperature and high-temperature co-calibration equipment to obtain the individual temperature characteristics of each sensor, and then proper temperature compensation is required.

## 3. Calibration and Analysis in High Temperature

According to Equation (10), for downhole pressure monitoring in high temperatures, ambient temperature has a significant impact on the measurement accuracy of the silicon piezoresistive pressure sensor. These temperature coefficients differ from manufacturer to manufacturer and even vary from batch to batch within the same manufacturer. As a result, in order to provide high accuracy and long-term stability for downhole pressure monitoring in high temperatures, it is critical to perform high-temperature and high-temperature co-calibration on each sensor and suggest an appropriate temperature compensation method.

### Design of Calibration Experiments

**Figure 2:**
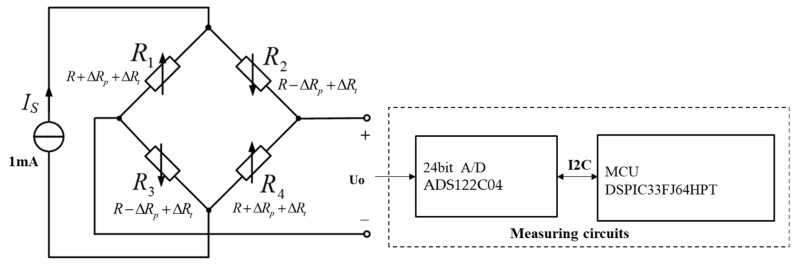
Pressure measuring systems.

Figure 2 depicts the whole pressure measuring system, including the pressure sensor and following high-temperature measurement circuits, such as signal conditioning, reference current source (1 mA), 24-bit A/D (ADS122C04), and 16-bit MCU (DSPIC33FJ64MC706HPT). To assure the accuracy of the overall pressure measuring system at various temperatures, the pressure sensor is calibrated in conjunction with the subsequent high-temperature measurement circuits.

The downhole 6LHP pressure sensors from Keller, Switzerland, with a measuring range of 0–60 MPa, were chosen for downhole pressure monitoring. These sensors are designed to withstand high-pressure and high-temperature environments for downhole pressure monitoring during oil & gas exploration and production, and they provide excellent long-term stability and high accuracy at temperatures up to 150 °C.

**Figure 3:**
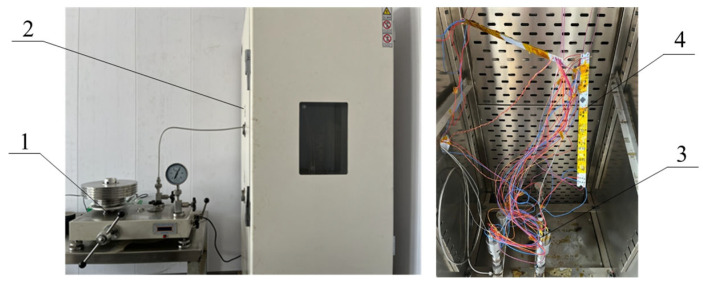
High-pressure and high-temperature co-calibration device. 1-dead weight pressure tester; 2-thermostat; 3-pressure sensors; 4-high-temperature measurement circuits.

A series of calibration tests were conducted on a high-pressure and high-temperature co-calibration device, shown in Figure 3. The device consists of a dead weight pressure tester with a measuring range of 0–100 MPa and an accuracy of 0.01% as reference pressure, a thermostat with temperature controls up to 200 °C and ±0.5 °C accuracy, and An A-class thin-film temperature sensor Pt1000 as reference temperature with a measuring range of −50~400 °C and an accuracy of ±0.15 °C.

**Figure 4:**
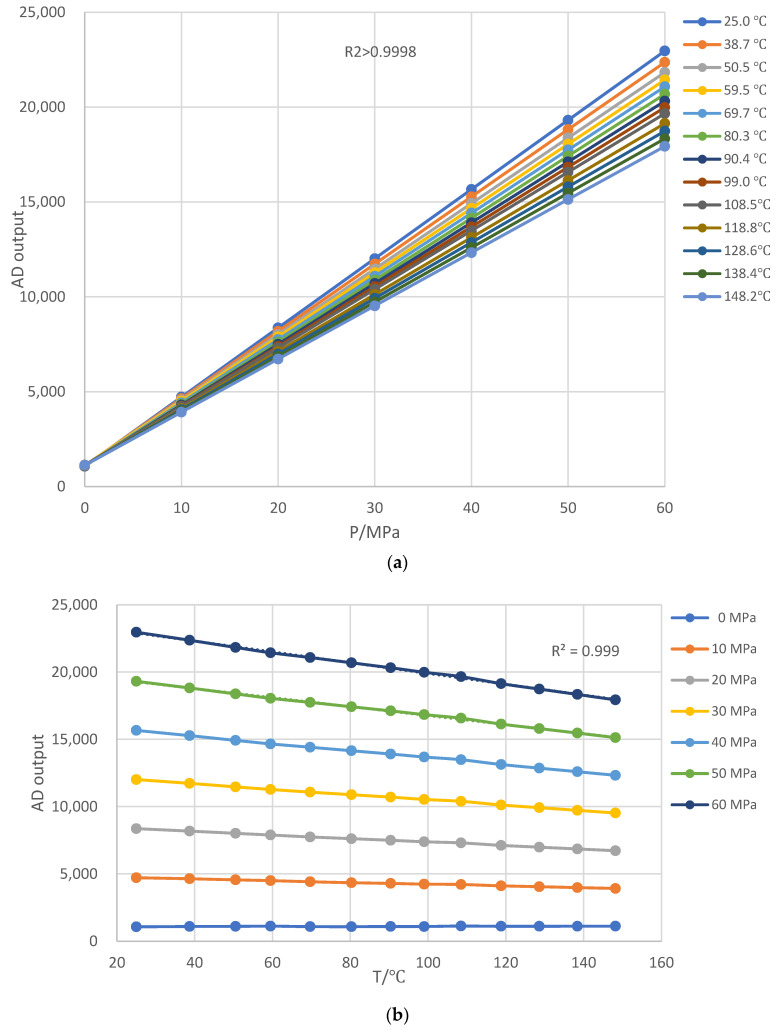
Test data of the pressure sensor. (**a**) AD-P curve respectively at each same temperature; (**b**) AD-T curve respectively at each same pressure.

Pressure sensors, temperature sensors, and circuit boards are all combined into a thermostat, and the pressure sensors are linked to the dead-weight pressure tester by hydraulic tubes. The experimental test range covers 0–60 MPa and room temperature to 150 °C. The temperature was raised from room temperature to 150 °C in 10 °C increments, and pressure was increased from 0 to 60 MPa in 10 MPa increments at each temperature point. Each test point was split into two tests: positive and negative strokes. The test results for one of the pressure sensors are reported in Table 1 and Figure 4.

Figure 4a shows that, when the temperature is constant, the AD output is positively linear with pressure P at the same temperatures, with R_2_ ≥ 0.9998, indicating compliance with Equation (2). Figure 4b shows that, when the pressure is constant, the AD output is nearly negatively linear with temperature T at the same pressure, with R_2_ ≥ 0.9989, indicating that there is only a slight non-linear between temperature and output in Equation (10). Its findings serve as the foundation for the following bilinear interpolation temperature compensation method.

**Figure 5:**
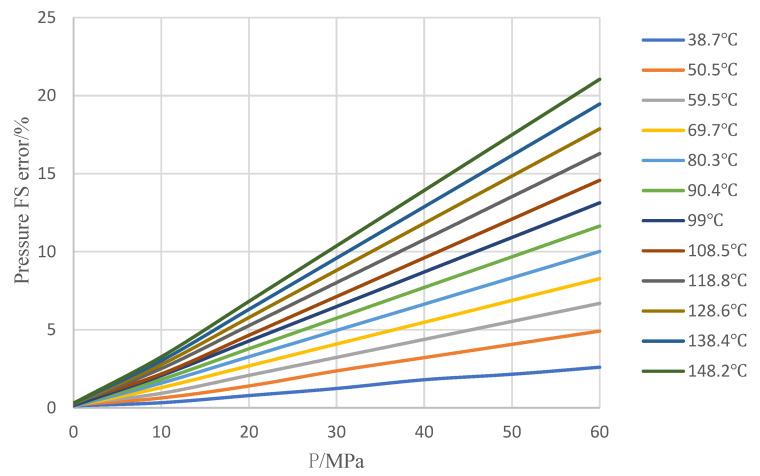
Full-scale error respectively at each same temperature.

Figure 5 depicts full-scale error respectively at each of the same temperatures, with 25 °C as the reference standard. When the temperature rises, the full-scale pressure error of the tested sensor is always positive with a maximum full-scale error close to 21.2% F.S. When the temperature is constant, the higher the pressure, the greater the error at each of the same temperatures. When the pressure is constant, the higher the temperature, the greater the error at each of the same pressures. As a result, a temperature compensation approach based on bilinear interpolation is proposed to provide excellent sensor accuracy for downhole pressure monitoring under high temperatures and pressures.

## 4. Bilinear Interpolation Temperature Compensation Method

Figure 4 shows that the AD value of the pressure measuring systems is positively linear with pressure P at the same temperature and nearly negatively linear with temperature T at the same pressure. Its result serves as the bias correction for the subsequent bilinear interpolation temperature compensation method. To reduce errors caused by the calibration process or other causes, AD-temperature data at the same pressure for each row of Table 1 were linearly fitted using least squares, and AD values were derived by interpolation of integer temperatures. After least squares fitting and interpolation, a new table with integer pressures and temperatures substituted Table 1, as illustrated in Table 2, making it easier for the microcontroller to implement.

Table 2 shows the link between the AD value, T, and P, which is more convenient for the MCU to implement. A bilinear interpolation method was presented to adjust for temperature effects on sensor accuracy, making it easier to implement on an MCU. The MCU can calculate the pressure value by performing bilinear interpolation on the temperature and pressure inputs in Table 2. It used two-dimensional segmentation and linear interpolation for temperature and pressure. The interpolation intervals for temperature and pressure are set at 10 °C and 10 MPa, respectively.

Let us imagine we are looking for the corrected pressure value. Using the bilinear interpolation temperature compensation approach, P may be represented as:

(11) $$P=f\left(x,y\right)$$

where *y* is the AD value observed by the pressure sensor, *x* is the measured value of temperature by Pt1000, and *P* is the compensated pressure value.

**Figure 6:**
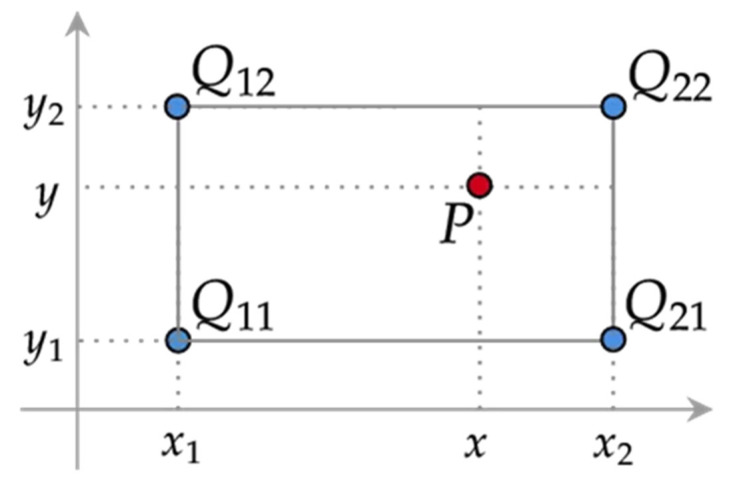
Bilinear interpolation temperature compensation method.

The first step would be to seek the nearest pressure values $f\left(Q_{11}\right)$, $f\left(Q_{12}\right)$, $f\left(Q_{21}\right)$ and $f\left(Q_{22}\right)$ from Table 2, as shown in Figure 6. Then the first interpolation calculation will be executed towards the temperature, as follows:

(12) $$\begin{array}{l} f\left(x,y_{1}\right)=\frac{x_{2}-x}{x_{2}-x_{1}}f\left(Q_{11}\right)+\frac{x-x_{1}}{x_{2}-x_{1}}f\left(Q_{21}\right)\\ f\left(x,y_{2}\right)=\frac{x_{2}-x}{x_{2}-x_{1}}f\left(Q_{12}\right)+\frac{x-x_{1}}{x_{2}-x_{1}}f\left(Q_{22}\right). \end{array}$$

where, $x_{1}$ and $x_{2}$ represent, respectively, the lower limit the upper and the limit temperature value within the temperature interpolation interval, $y_{1}$ and $y_{2}$ are respectively the lower limit and the upper limit of AD value for the pressure interpolation interval. 

Following that, the second interpolation calculation will be executed directed toward the pressure direction, the compensated pressure value P can be obtained, as follows:

(13) $$P=f(x,y)=\frac{1}{\left(x_{2}-x_{1}\right)\left(y_{2}-y_{1}\right)}\left[\begin{array}{cc} x_{2}-x & x-x_{1} \end{array}\right]\left[\begin{array}{cc} f\left(Q_{11}\right) & f\left(Q_{12}\right)\\ f\left(Q_{21}\right) & f\left(Q_{22}\right) \end{array}\right]\left[\begin{array}{l} y_{2}-y\\ y-y_{1} \end{array}\right]$$

**Figure 7:**
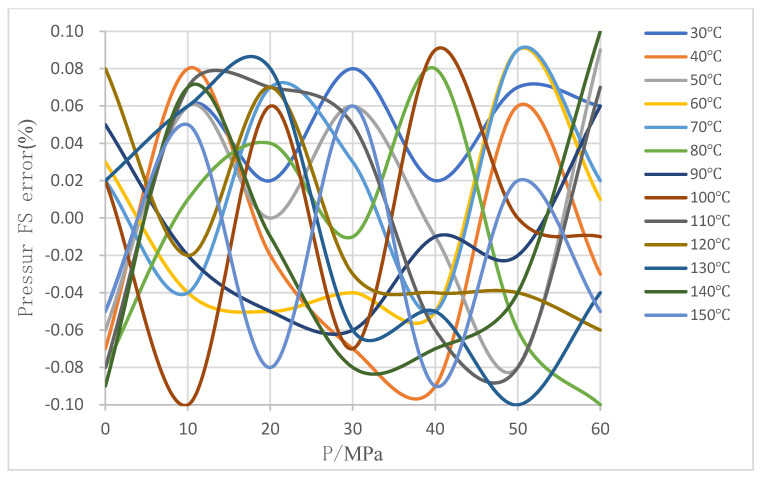
Full-scale error after bilinear interpolation compensation.

A series of experiments covering the complete measurement range were conducted to assess the effectiveness of the suggested temperature compensation approach. The test results are displayed in Figure 7.

Compare with uncompensated measurement accuracy, shown in Figure 5, after applying the bilinear interpolation method for temperature compensation, it improves significantly the overall measurement accuracy of the tested pressure sensor from 21% F.S. to 0.1% F.S., which can meet high accuracy demand for downhole pressure monitoring in high temperature and high-pressure conditions, shown in Figure 7.

## 5. Conclusions

In this study, a temperature compensation method based bilinear interpolation on for high- accuracy piezoresistive pressure sensors suitable for downhole high-temperature and high-pressure environments is proposed, which significantly improves the accuracy of the pressure measurement while also reducing the computational complexity of the MCU compensation algorithm.

To assure the accuracy of the entire pressure measuring system at various temperatures, the pressure sensor is calibrated alongside the succeeding high-temperature measuring circuits, which include signal conditioning, current source, A/D, and MCU. Calibration and experimental tests were performed over a range of 0 to 60 MPa, and between room temperature and 150 °C, in order to calibrate and evaluate the measurement accuracy of the pressure measuring system before and after temperature adjustment. The following conclusions can be drawn:

(1) The AD output of the tested pressure measurement system is positively linear with pressure P at each of the same temperatures with R^2^ ≥ 0.9998, but practically negatively linear with temperature T at each of the same pressure with R^2^ ≥ 0.9999. Its findings serve as the foundation for the following bilinear interpolation temperature compensation method. The full-scale pressure error of the tested pressure measurement system is always positive, and the bigger the error at each pressure, with a maximum full-scale error of around 21.2% F.S.
(2) To ensure high accuracy and long-term stability for downhole pressure monitoring in high temperatures, we propose a temperature compensation method based on bilinear interpolation for high-accurate piezoresistive pressure sensors suitable for downhole high-temperature and high-pressure environments. The test results revealed that the proposed method significantly improves the overall measurement accuracy of the tested pressure sensor from 21.2% F.S. to 0.1% F.S. In addition, it reduces the MCU computational complexity of the compensation model, meeting the high accuracy demand for downhole pressure monitoring in high temperatures and high pressure.
(3) For downhole pressure monitoring in high-temperature and high-pressure environments, ambient temperature has a major impact on the silicon piezoresistive pressure sensor’s measurement accuracy. The temperature coefficients shown above differ from manufacturer to manufacturer and even vary from batch to batch within the same manufacturer. When the sensor is used for common temperature and low accuracy applications, ambient temperature effects are so small as to be negligible. However, to ensure high accuracy and long-term stability for downhole pressure monitoring in high temperatures, each sensor must undergo high-temperature and high-temperature co-calibration, as well as develop the corresponding temperature compensation model based on bilinear interpolation separately. However, the cost of calibration equipment and labor is high, even higher than the value of the sensor itself, which requires us to weigh accuracy against the cost for different downhole pressure monitoring requirements.

## Figures and Tables

**Table 1:** Test data of the pressure sensor.

	T(°C)	25	38.7	50.5	59.5	69.7	80.3	90.4	99	108.5	118.8	128.6	138.4	148.2
P(MPa)	AD													
0		1069	1095	1107	1120	1085	1083	1093	1091	1129	1111	1113	1116	1119
10		4718	4640	4561	4506	4417	4350	4298	4240	4218	4115	4051	3986	3922
20		8367	8185	8016	7892	7750	7618	7502	7389	7308	7120	6988	6856	6724
30		12,015	11,730	11,470	11,277	11,082	10,886	10,707	10,538	10,397	10,125	9926	9726	9527
40		15,664	15,275	14,925	14,663	14,415	14,153	13,911	13,687	13,486	13,130	12,863	12,596	12,329
50		19,313	18,820	18,379	18,049	17,748	17,421	17,116	16,836	16,575	16,135	15,801	15,466	15,132
60		22,961	22,366	21,834	21,435	21,080	20,688	20,320	19,985	19,664	19,140	18,738	18,336	17,935

**Table 2:** Test data after fitting and interpolating.

	T(°C)	30	40	50	60	70	80	90	100	110	120	130	140	150
P(MPa)	AD													
0		1086	1089	1092	1094	1097	1100	1103	1105	1108	1111	1114	1117	1119
10		4689	4625	4560	4496	4431	4367	4302	4238	4173	4109	4044	3980	3915
20		8293	8161	8029	7897	7766	7634	7502	7370	7238	7107	6975	6843	6711
30		11,896	11,697	11,498	11,299	11,099	10,900	10,701	10,502	10,303	10,104	9905	9706	9507
40		15,499	15,233	14,966	14,700	14,433	14,167	13,901	13,634	13,368	13,102	12,835	12,569	12,303
50		19,103	18,769	18,436	18,102	17,768	17,435	17,101	16,768	16,434	16,100	15,767	15,433	15,099
60		22,706	22,305	21,904	21,503	21,102	20,702	20,301	19,900	19,499	19,098	18,697	18,296	17,895

## Data Availability

The data presented in this study are available on request from the corresponding author due to privacy.
